# Pre-hospital treatment of bee and wasp induced anaphylactic reactions: a retrospective study

**DOI:** 10.1186/s13049-016-0344-y

**Published:** 2017-01-14

**Authors:** Athamaica Ruiz Oropeza, Søren Mikkelsen, Carsten Bindslev-Jensen, Charlotte G. Mortz

**Affiliations:** 1 Department of Dermatology and Allergy Center, Odense Research Center for Anaphylaxis (ORCA), Odense University Hospital, Sdr. Boulevard 29, Odense, DK-5000 Denmark; 2 Department of Anesthesiology and Intensive Care Medicine, Mobile Emergency Care Unit, Odense University Hospital, Odense, Denmark

**Keywords:** Anaphylaxis, Pre-hospital treatment, Severity

## Abstract

**Background:**

Bee and wasp stings are among the most common triggers of anaphylaxis in adults representing around 20% of fatal anaphylaxis from any cause. Data of pre-hospital treatment of bee and wasp induced anaphylactic reactions are sparse. This study aimed to estimate the incidence of bee and wasp induced anaphylactic reactions, the severity of the reactions and to correlate the pre-hospital treatment with the severity of the anaphylactic reaction.

**Methods:**

Retrospective and descriptive study based on data from the Mobile Emergency Care Units (MECUs) in the Region of Southern Denmark (2008 only for Odense and 2009–2014 for the whole region). Discharge summaries with diagnosis related to anaphylaxis according to the International Classification of Diseases 10 (ICD-10) were reviewed to identify bee and wasp induced anaphylactic reactions. The severity of the anaphylactic reaction was assessed according to Sampson’s severity score and Mueller’s severity score. Treatment was evaluated in relation to administration of adrenaline, glucocorticoids and antihistamine.

**Results:**

We identified 273 cases (Odense 2008 *n =* 14 and Region of Southern Denmark 2009–2014 *n =* 259) of bee and wasp induced anaphylaxis. The Incidence Rate was estimated to 35.8 cases per 1,000,000 person year (95% CI 25.9–48.2) in the Region of Southern Denmark during 2009–2014. According to Sampson’s severity score, 65% (*n =* 177) of the cases were graded as moderate to severe anaphylaxis (grade 3–5). Almost one third of cases could not be graded according to Mueller’s severity score. Adrenaline was administrated in 54% (96/177) of cases with moderate to severe anaphylaxis according to Sampson’s severity score, compared to 88% receiving intravenous glucocorticoids (*p < 0.001*) and 91% receiving intravenous antihistamines (*p < 0.001*). Even in severe anaphylaxis (grade 5) adrenaline was administered in only 80% of the cases.

**Conclusion:**

Treatment with adrenaline is not administered in accordance with international guidelines. However, making an assessment of the severity of the anaphylactic reaction is difficult in retrospective studies.

## Background

Anaphylaxis is a severe, life-threatening generalized hypersensitivity reaction [1]. Population-based studies have reported a prevalence of bee and wasp induced anaphylaxis ranging from 0.3 to 8.9% in adults and from 0.15 to 0.8% in children [2, 3]. Several studies have reported bee and wasp stings to be the most common trigger of anaphylactic reactions in adults [4, 5]. The incidence of fatalities due to bee and wasp induced anaphylactic reactions ranges from 0.3 to 0.48 cases per one million inhabitants per year, representing around 20% of fatal anaphylaxis from any cause [2, 3]. In Denmark, bee and wasp induced anaphylactic reactions were reported to be 40% of anaphylactic cases pre-hospital level in the 1980s [6] and the number of fatalities related to this condition in Denmark was in the 1980s estimated to one per year or 0.25 cases per 1,000,000 inhabitants per year [7, 8]. No recent data are available in Denmark.

The first line treatment of anaphylaxis is intramuscular (IM) administered adrenaline [9, 10]. However, a recent German study has shown that adrenaline is administered in only 22.7% of anaphylaxis cases in the emergency setting and only in 29.6% of cases with severe anaphylaxis [11]. This is in line with previous studies in the emergency department [12–14], reflecting poor concordance between guidelines and actual treatment in the acute situation in the emergency settings [11, 15].

Bee and wasp induced anaphylactic reactions are often treated at a pre-hospital level. However, data of pre-hospital treatment of anaphylaxis are sparse [5, 16, 17].

With this study, we aimed 1) to estimate the incidence of bee and wasp induced anaphylactic reactions at pre-hospital level in the Region of Southern Denmark (2009–2014) and 2) the severity of the reactions, and 3) to correlate pre-hospital treatment to the severity of the anaphylactic reaction.

## Methods

This is a retrospective and descriptive study based on data from the Mobile Emergency Care Units (MECUs) in the Region of Southern Denmark during the period January 1^st^, 2008 to December 31^st^, 2014. During 2008 the MECU covered only the city of Odense with a population of 187,138 persons. From 2009, however, the emergency medical system in the Region of Southern Denmark was expanded to consist of six MECUs servicing the entire population of 1,201,366 persons and covering an area of 122.00 km^2^. The Mobile Emergency Care Unit (MECU) operates as part of a three-tiered system, in which the MECU supplements an ordinary ambulance manned with two emergency medical technicians (EMTs) or a paramedic and an ambulance. The MECU consists of a rapid response car manned with a specialist in anesthesiology and an EMT [18].

Following each MECU run, the attending anesthesiologist documents the mission in the MECU database including registration of tentative diagnosis according to the International Classification of Diseases 10 (ICD-10), vital signs, treatment administered and outcome of mission [18, 19]. Discharge summaries with ICD-10 diagnosis: T63.4 Venom of other arthropods (insect bite or sting, venomous), T78.2 Anaphylactic shock, unspecified, T78.3 Angioneurotic edema, T78.4 Allergy, unspecified and I46.0 Cardiac arrest with successful resuscitation, were extracted from the MECU database and reviewed in order to identify the cases of bee and wasp induced anaphylaxis.

Discharge summaries with anaphylaxis induced by other triggers than bee or wasp were excluded, as well as the summaries without any description of symptoms since these could not be used to assess the severity of the anaphylactic reactions. In the case of patients encountered more than once during the study period, only the first contact was included, but the characteristics of the subsequent contacts were described.

The severity of the anaphylactic reaction was assessed according to two severity score systems. We used Sampson’s severity score (Table 1) [20] which was chosen because it is the score that is used routinely in the Allergy Center. It comprises a 5 grades scoring system that ranges from mild (grade 1) to severe symptoms of anaphylaxis (grade 5). Furthermore, we used Mueller’s severity score (Table 2) [21], which is a 4 grades system designed for the assessment of insect stings induced anaphylaxis. Treatment was evaluated in relation to administration of adrenaline IM, adrenaline intravenous (IV) or combination of both, glucocorticoids IV and antihistamines IV. Data on the population living in the city of Odense in 2008 and in the Region of Southern Denmark during 2009–2014 were collected at the StatBank Denmark website (http://www.statistikbanken.dk; accessed November 2016).

**Table 1:** Grading of anaphylaxis according to severity of clinical symptoms. Sampson 2003

Grade	Skin	GI Tract	Respiratory Tract	Cardiovascular	Neurological
1	Localized pruritus, flushing, urticaria, angioedema	Oral pruritus, oral “tingling”, mild lip swelling			
2	Generalized pruritus, urticaria, angioedema	Any of the above, nausea and/or emesis x’s 1	Nasal congestion and/or sneezing		Change in activity level
3	Any of the above	Any of the above, plus repetitive vomiting	Rhinorrhea, marked congestion, **sensation of throat pruritus or tightness**	Tachycardia (increase >15 beats/min)	Change in activity level plus anxiety
4	Any of the above	Any of the above plus diarrhea	Any of the above, **hoarseness, “barky” cough, difficulty swallowing, dyspnea, wheezing, cyanosis**	Any of the above, **dysrhythmia and/or mild hypotension**	“Light headedness” feeling of “*pending doom*”
5	Any of the above	Any of the above, loss of bowel control	Any of the above, **respiratory arrest**	**Severe bradycardia and/or hypotension or cardiac arrest**	**Loss of consciousness**

All symptoms are not mandatory. The severity score should be based on the organ system most affected, e.g., if grade 3 respiratory symptoms are present but only grade 1 GI symptoms, then the anaphylaxis severity score would be “grade 3”. Boldface letters are absolute indications for the use of epinephrine; use of epinephrine with other symptoms will depend on patient’s history

**Table 2:** Grading of anaphylaxis according to severity of clinical symptoms. Mueller 1966

Grade	Signs and Symptoms
1 Slight general reaction	Generalized urticaria, itching, malaise and anxiety
2 General reaction	Any of the above plus two or more of following: generalized edema; constriction in chest; wheezing; abdominal pain; nausea and vomiting; and dizziness
3 Severe general reaction	Any of the above plus two or more of following: dyspnea; dysphagia; hoarseness or thickened speech; confusion; and feeling of impending disaster
4 Shock reaction	Any of the above plus two or more of following: cyanosis; fall in blood pressure; collapse incontinence; and unconsciousness

### Statistics

Baseline characteristics are presented as numbers and percentages. The Incidence Rate (IR) of anaphylaxis was calculated for the Region of Southern Denmark during 2009–2014 as the number of patients having anaphylaxis for the first time per 1,000,000 person year with the correspondent 95% confidence interval based on a binomial distribution (CI 95%). Comparisons were made by *χ* ^2^-based table analysis. Statistical significance was defined as *p < 0.05*. Statistical analysis was performed with Stata IC 14.0 (Stata Corporation LP®, Texas, USA).

### Ethics

The study was approved by the Danish National Board of Health (J. no. 3–3013–911/1/) and the Data Protection Agency (J. no. 15/4004). Approval by an Ethics Committee or informed consent is not required for register-based research in Denmark.

## Results

**Fig. 1:**
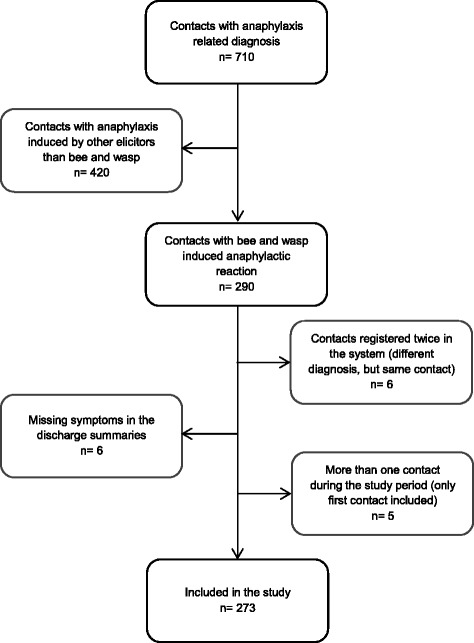
Flow diagram of patients with bee and wasp induced anaphylactic reaction attended by the MECU during 2008-2014

We reviewed 710 discharge summaries. Two hundred and seventy-three summaries (39%) fulfilled the inclusion criteria of bee and wasp induced anaphylactic reactions. Numbers of individuals at each stage of the study and reasons for exclusion are presented in Fig. 1. The study population included 88 women and 185 men with a median age of 53 years; 20 children (0–17 years) and 253 adults (18–91 years). Most cases were seen between May and September. The Incidence Rate of bee and wasp induced anaphylaxis was calculated in 35.8 cases per 1,000,000 person year (95% CI 25.9–48.2) in the Region of Southern Denmark during 2009–2014 (*n =* 259).

Three deaths were registered during the study period: two men (46 and 63 years old) and one woman (80 years old). In these three cases the symptoms were described as follows: one had loss of consciousness, respiratory and cardiac arrest; one was stung in the mouth with severe swelling and asystole; and the third one had hypotension and loss of consciousness. Only the first patient received treatment with adrenaline IV, glucocorticoids IV and antihistamines IV. The other 2 patients did not receive any treatment as they were found dead at the MECU arrival.

The 3 deaths represented 1% of our study population and a mortality rate of 0.4 cases per 1,000,000 person year was calculated in the study period.

The group of patients with multiple encounters included three men and two women. All were seen twice in the period and had moderate to severe anaphylaxis (grade 3–5) at the first contact and received treatment with adrenaline IM, glucocorticoids IV and antihistamines IV. At the second contact four of the five patients had moderate to severe symptoms while one had grade 2 symptoms (generalized erythema and edema of the tongue). At the second contact all cases were treated with glucocorticoids IV and antihistamines IV. Adrenaline IM was administered to the four patients with moderate to severe symptoms.

### Severity of the anaphylactic reaction

**Fig. 2:**
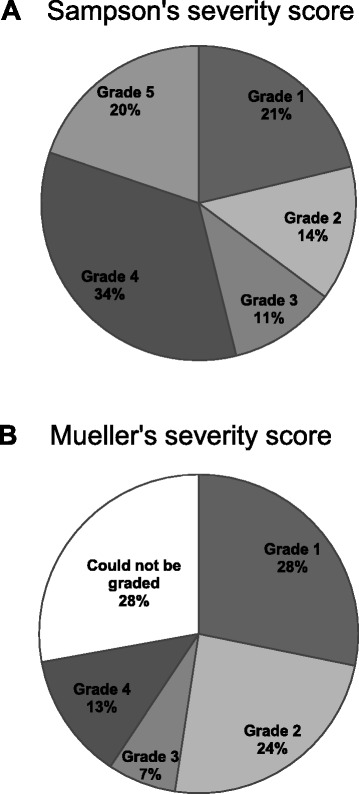
Severity of the anaphylactic reaction evaluated by Sampson’s severity score (**a**) and Mueller’s severity score (**b**) (*n =* 273)

All cases of bee and wasp induced anaphylactic reactions could be graded according to Sampson’s severity score; 177 (65%) of the cases were graded as moderate to severe anaphylaxis (grade 3 to 5) (Fig. 2). Using Mueller’s score, 76 (28%) of the cases could not be graded (distributed in Sampson’s severity score as grade 1 (*n =* 51), grade 2 (*n =* 3), grade 3 (*n =* 4), grade 4 (*n =* 12) and grade 5 (*n =* 6)). Therefore, Sampson’s severity score was used for further analysis.

Among the 20 children, most had local symptoms (grade 1, *n =* 9) or had generalized skin symptoms (grade 2, *n =* 5), while 6 (30%) presented with moderate to severe anaphylaxis. In the group of adults moderate to severe anaphylaxis was observed in 171 (68%) patients.

### Treatment in relation to the severity of the reaction

**Fig. 3:**
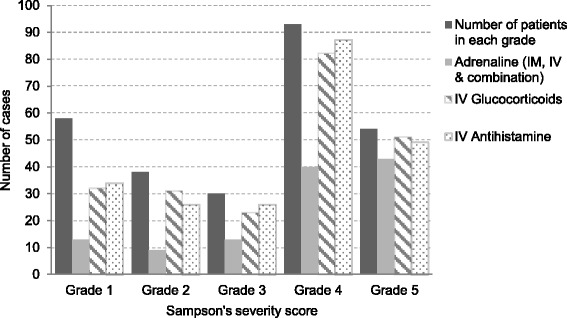
Treatment administrated by the MECU in relation to the severity of the anaphylactic reaction

The administration of adrenaline, glucocorticoids and antihistamines according to the severity of the anaphylactic reaction is shown in Fig. 3.

Patients with moderate to severe anaphylaxis (*n =* 177) received treatment with adrenaline in 54% (96/177) of the cases (IM 78%, IV 8%, combination of both 14%), compared to 88% (156/177) receiving intravenous glucocorticoids (*p < 0.001*) and 91% (162/177) receiving intravenous antihistamines (*p < 0.001*). Including only the more severe reactions (grade 4 to 5) the results did not change; hence, 56% (83/147) of the patients in grade 4–5 and, 80% (43/54) of the cases in grade 5, were treated with adrenaline, respectively.

The proportion of cases with moderate to severe anaphylaxis treated with adrenaline was stable during the study period (2008–2014) except for the year 2012, where 26% of the cases received treatment with adrenaline (data not shown). This was significantly lower compared both to the previous years (*p < 0.02*) and the following years (*p < 0.02)*. The results were not changed by only including grade 4 to 5.

## Discussion

This is the first observational, retrospective study assessing bee and wasp induced anaphylactic reactions at pre-hospital level in Denmark. Our study reveals that moderate to severe anaphylaxis was observed in 65% of anaphylactic patients treated by the MECU in the Region of Southern Denmark. Furthermore, our results showed that treatment with adrenaline in cases with moderate to severe anaphylaxis was performed in only half of the cases while most cases were treated with glucocorticoids and antihistamines.

A few other studies have evaluated the severity of anaphylaxis induced by bee and wasp sting in the emergency room [22, 23]. A recent study from Belgium shows that 67% of bee and wasp induced anaphylactic reactions were grade 4–5 using Sampson’s severity score in line with our pre-hospital findings [22], whereas a retrospective study in a Swiss emergency department showed that only 6% were graded as moderate to severe anaphylaxis using Mueller’s severity score [23]. Using Mueller’s scoring system, we were unable to classify 28% of the anaphylaxis cases in our retrospective study. Using the Sampson’s severity score, however, all patients could be classified. Sampson’s severity score includes 5 grades from mild to severe anaphylaxis, includes local symptoms and allowed scoring according to single symptoms such as hypotension [20]. In Mueller’s severity score, symptoms described in the milder grades of anaphylaxis are mandatory to reach a higher severity grade [21]. This means that in retrospective studies, when most often only the most severe symptoms such as hypotension or respiratory symptoms are described in details, Mueller’s severity score is less suitable for evaluation. In our study 23% (22/76) of those that could not be classified by Mueller’s severity score had moderate to severe anaphylaxis according to the Sampson severity score. This supports an issue previously discussed by Bilò et al [3] and Golden et al [24]. They state that some of the frequently used severity scores as Mueller’s [21] and Ring’s [25] do not consider the possible absence of cutaneous symptoms in the reaction. This risks misclassification of reactions with isolated hypotension, as well reactions with symptoms from other organ systems than the skin, contributing to a possible under-diagnosis of anaphylaxis [26].

In line with previous studies of anaphylaxis at pre-hospital level [16, 27, 28] and in the emergency room [11], our study shows that adrenaline was administered in only 54% of patients with moderate to severe anaphylaxis whereas antihistamines and glucocorticoids were administrated in nearly all cases. Not all symptoms in Sampson’s severity score grade 3 require treatment with adrenaline. However, including only the more severe reactions (Sampson’s grade 4–5) did not change the result (54% versus 56%) and including only reactions classified as Sampson’s grade 5, still only 80% received adrenaline. The last finding supports an older pre-hospital study in Scandinavia on severe anaphylactic reactions managed by anesthesiologist-staffed ambulance helicopters [5], showing that adrenaline is still administered in only 78–80% of the severe anaphylaxis cases. This is in contrast with the current international guidelines for anaphylaxis treatment [9, 10]. However, the low mortality rate observed in this study, in line with previous Danish estimates [7, 8] and international studies [2, 3], does not seem to reflect the lack of adrenaline administration.

Various factors may explain the reluctance to use adrenaline in the acute treatment of moderate to severe anaphylaxis. Firstly, the MECU is served by a specialist in Intensive Care Medicine which closely observes the patient and the vital parameters. The real clinical situation could be different than the data obtained from the files retrospectively suggests. The recording of a respiration rate above the normal rate could retrospectively be considered a case of respiratory insufficiency, while it in fact might simply be hyperventilation caused by fear and anxiety. Furthermore, in the acute situation, treatment of respiratory symptoms with β_2_-agonist inhalation may have resolved the symptoms and in some cases the patient may recover spontaneously even without administration of adrenaline. Also, in some cases, a pre-existing cardiovascular condition may have affected the decision whether to use adrenaline or not.

The appropriateness of administration of adrenaline in patients with anaphylaxis has recently been discussed in one study in the emergency care setting where even though more than 60% of the patients with anaphylaxis did not receive adrenaline, the allergists-immunologists found that the management of the reaction was appropriate in 98% of the cases [29].

Despite this, it is necessary to comprehend the importance of the treatment with adrenaline due its effect on α-1, β-1 and β-2 receptors, reversing the life-threatening symptoms of anaphylaxis [10], but at the same time the assessment of the physician on charge is determinant and it should not be underrated.

Among the limitations of this study are: The retrospective design; the classification of the reactions in the scoring systems was not performed by the emergency physicians in the acute settings; the assessment was done retrospectively based on the described symptoms in the discharge summaries; the discharge summaries may not necessarily include all symptoms as they may only include the most severe symptom, and the course of the reaction was not always clearly described. This may bias the results. Furthermore, data including evaluation of Serum-tryptase (both during the reaction and at basic), and specific Serum-IgE to venom were not available as well as co-morbidity such as mastocytosis and co-factors such as NSAID or exercise.

The strengths of this study are the size of the population, the long time span (2008–2014) and the fact that the MECU includes treatment at pre-hospital level, where the majority of bee and wasp induced anaphylactic reactions take place. Also, the character of the emergency care system with MECUs being dispatched to all perceived severely ill patients in the region ensures that most of the patients being affected by bee and wasp induced anaphylactic reactions were registered [30]. Likewise, the Danish Civil Registration number system [31] ensures that the discharge summary of every patient encountered by the MECU can be accessed in the patient’s in-hospital medical files.

## Conclusion

Assessment of the severity of the anaphylactic reaction is difficult in retrospective studies due the lack of description of all signs and symptoms.

Although adrenaline is first choice of drug in cases of anaphylaxis and thus of higher priority than glucocorticoids and antihistamines, this was administered to a lesser extent than the two latter drugs. There seems to be a potential for improving prehospital physicians adherence to guidelines.

## Abbreviations

EMT: Emergency medical technicians
ICD-10: International classification of diseases 10
IM: Intramuscular
IV: Intravenous
MECU: Mobile emergency care unit
